# Acute promyelocytic leukaemia: population-based study of epidemiology and outcome with ATRA and oral-ATO from 1991 to 2021

**DOI:** 10.1186/s12885-023-10612-z

**Published:** 2023-02-10

**Authors:** Harinder Gill, Radha Raghupathy, Carmen Y.Y. Lee, Yammy Yung, Hiu-Tung Chu, Michael Y. Ni, Xiao Xiao, Francis P. Flores, Rita Yim, Paul Lee, Lynn Chin, Vivian W.K. Li, Lester Au, Wing-Yan Au, Edmond S.K. Ma, Diwakar Mohan, Cyrus Rustam Kumana, Yok-Lam Kwong

**Affiliations:** 1 Department of Medicine, School of Clinical Medicine, LKS Faculty of Medicine, The University of Hong Kong, Hong Kong SAR, China; 2 School of Public Health, LKS Faculty of Medicine, The University of Hong Kong, Hong Kong SAR, China; 3 Healthy High Density Cities Lab, HKUrbanLab, the University of Hong Kong, Hong Kong SAR, China; 4 The State Key Laboratory of Brain and Cognitive Sciences, the University of Hong Kong, Hong Kong SAR, China; 5 Blood-Med Clinic, Hong Kong SAR, China; 6 Department of Pathology, Hong Kong Sanatorium and Hospital, Hong Kong SAR, China; 7 Division of Global Disease Epidemiology and Control, Department of International Health, John Hopkins Bloomberg School of Public Health, Baltimore, USA; 8 Department of Medicine, Queen Mary Hospital, Pokfulam Road, Professorial Block, Hong Kong, China

**Keywords:** Acute promyelocytic leukaemia, Epidemiology, Oral arsenic trioxide, Early deaths, Survivals, Second primary cancers

## Abstract

**Background:**

The epidemiology and treatment of acute promyelocytic leukaemia (APL) are changing. We have incorporated oral arsenic trioxide (oral-ATO) into induction/maintenance.

**Methods:**

Newly-diagnosed APL from 1991 to 2021 divided into three 10-year periods were studied to define its epidemiology and how oral-ATO impacted on its outcome. Primary endpoints included APL incidence, early deaths (ED, first 30 days), and overall survival (OS). Secondary endpoints included post-30-day OS, relapse-free survival (RFS), and incidence of second cancers.

**Results:**

APL occurred in 374 males and 387 females at a median age of 44 (1–97) years. Annual incidences increased progressively, averaging 0.32 per 100,000 people. All-trans retinoic acid (ATRA)-based and oral-ATO-based regimens were used in 469 and 282 patients. There were 144 EDs, occurring almost exclusively in ATRA-based inductions (N = 139), being more with males, age > 50 years, leucocyte > 10 × 10^9^/L, diagnosis during 1991–2009 and fewer with oral-ATO-based regimens. After a median of 75 (interquartile range: 14–161) months, 5-year and 10-year OS were 68.1% and 63.3%, inferior with males, age > 50 years, leucocyte > 10 × 10^9^/L, high-risk Sanz score and superior with oral-ATO-based regimens. Factoring out EDs, 5-year and 10-year post-30-day OS were 84.0% and 78.1%, inferior with males and superior with oral-ATO-based regimens. In 607 CR1 patients, the 5-year RFS was 83.8%, superior with diagnosis in 2010–2021 and oral-ATO-based regimens. Second cancers developed in 21 patients, unrelated to oral-ATO-based regimens.

**Conclusions:**

There was an increasing incidence of APL, and all survivals were superior with the use of oral-ATO-based regimens. This study formed part of the Acute Promyelocytic Leukaemia Asian Consortium Project (ClinicalTrials.gov identifier: NCT04251754).

**Supplementary Information:**

The online version contains supplementary material available at 10.1186/s12885-023-10612-z.

## Introduction

Acute promyelocytic leukaemia (APL) arises from t(15;17)(q24;21) and *PML*::*RARA* gene fusion [1]. On presentation, patients are at risk of life-threatening bleeding, due to thrombocytopenia and a characteristic coagulopathy. The introduction of all-trans retinoic acid (ATRA), together with vigorous supportive care, has much improved the outlook. First complete remission (CR1) rates of > 90%, and long-term survivals of > 85%, could be achieved in patients treated with ATRA and chemotherapy [2–5]. The advent of arsenic trioxide (ATO) has significantly changed frontline protocols, with most current induction regimens incorporating intravenous ATO (i.v.-ATO) with ATRA ± chemotherapy, which have resulted in CR rates of 90–100% and long-term survivals of 86–97% [6–10].

We formulated an oral preparation of ATO (oral-ATO), [11] and showed that it was efficacious for APL in first relapse (R1), inducing CR2 in more than 90% of patients [12, 13]. To prevent relapses, we moved oral-ATO forward to CR1 maintenance, showing that it was safe and resulted in favorable survivals [14]. From 2013, oral-ATO has been advanced into frontline protocols [15, 16].

While therapeutic strategies are evolving, the demographics and epidemiology of APL are also changing. Ethnic differences in the incidences of APL are emerging, together with a shift in the peak age at diagnosis to the elderly [17–19]. Furthermore, the curability of APL brings into focus the long-term safety of treatment, especially the development of second primary cancers [20, 21]. Hence, a critical appraisal of the management approach of APL is warranted, so that resources can be appropriately re-deployed in light of its changing epidemiology, and the most efficacious and least toxic regimens can be offered.

To address these issues, we evaluated the epidemiological landscape and treatment results of APL in the past three decades, with a view to improving further the service for our patients, and examining the impact of oral-ATO on treatment outcome and long-term safety.

## Patients and methods

***Patients***. In Hong Kong, over 95% of the population of 7.5 million people receive treatment in government hospitals. There are eighteen government hospitals with haematology specialist care. Patients with newly-diagnosed APL presenting to these hospitals in a 30-year period (January 1, 1991 to March 31, 2021) were identified with the Clinical Data Analysis and Reporting System (CDARS), which computerized all patient information. Data retrieved included the diagnosis according to International Classification of Diseases coding, Ninth Revision (ICD-9), date of first admission, sex, date of birth, date of death if applicable, and causes of death according to ICD-9 coding. The Laboratory Information System within CDARS was used to retrieve the presentation blood count. Specific information on APL, including the morphology, karyotype, and reverse transcription polymerase chain reaction for *PML-RARA*, was collected from individual patient records. The pathology reports were scrutinized for confirmation of the diagnosis of APL according to standard morphologic/karyotypic/molecular criteria [1]. The study was approved by the Institutional Review Board of the University of Hong Kong/Hong Kong West Cluster (UW 19–873), and formed part of the Acute Promyelocytic Leukaemia Asian Consortium Project (ClinicalTrials.gov identifier: NCT04251754).

***Treatment for newly-diagnosed patients.*** Therapeutic strategies for patients ≥ 18 years old varied according to the time periods of treatment (Supplemental file 1). From 1991 to 2012, standard induction comprised ATRA (45 mg/m^2^/day for 42 days) with daunorubicin (50 mg/m^2^/day for 3 days) (ATRA-based). Daunorubicin was omitted in patients aged 70 or above or those with severe co-morbidities. In CR1 patients, two maintenance strategies, ATRA/methotrexate/6-mercaptopurine and ATRA/oral-ATO/ascorbic acid (AAA was available from 2001 onwards), were adopted at the discretion of the attending physicians. Patients given AAA maintenance were referred to Queen Mary Hospital, the only hospital where any form of ATO was available (oral-ATO was used exclusively). From 2013 onwards, induction regimens with oral-ATO (10 mg/day for 42 days), ATRA (45 mg/m^2^/day for 42 days) with daunorubicin (50 mg/m^2^/day for 3 days) were administered at Queen Mary Hospital in clinical trials (oral-ATO-based) (Supplemental file 1) [15]. Again, daunorubicin was omitted in patients aged 70 or above or those with severe co-morbidities. In patients < 18 years old, the International Consortium for Childhood Acute Promyelocytic Leukaemia Study 01 protocol [22] was used throughout the study period (Supplemental file 1). Induction regimen comprised ATRA (25 mg/m^2^/day for at least 28 days) and Idarubicin (12 mg/m^2^/day on days 3, 5 and 7).

***Treatment for relapsed patients.*** Re-induction with ATRA, oral-ATO and idarubicin was available since 2002 in clinical trials, and patients were referred to and treated at Queen Mary Hospital [23]. Autologous or allogeneic haematopoietic stem cell transplantation was not routinely performed in CR2, and performed for selected patients at CR3 [23]. Patients < 18 years old were treated with re-induction with i.v.-ATO or oral-ATO, together with ATRA and chemotherapy [13].

***Endpoints and definitions***. Primary endpoints were the incidence of APL, early deaths (ED, defined as death within the first 30 days of presentation), 30-day survival (defined as time from presentation to ED, censoring at day 30), and the overall survival (OS, defined as time from presentation to death or last follow-up). Secondary endpoints included post-30-day OS (defined as time from day 31 to death or last follow-up), relapse-free survival (RFS, defined as time from CR1 to R1, death or last follow-up) and the incidence of second primary cancers. Data for the analysis of OS, post-30 day OS and RFS were censored on November 30, 2021.

***Statistical analyses.*** Categorical variables were analyzed with chi-square test and continuous variables with non-parametric tests. Survivals (30-day survival, OS, post-30-day survival, RFS) were analyzed with the Kaplan-Meier method, difference between groups determined with the log-rank test and Cox proportional hazard model. Prognostic impacts on survivals were evaluated for the following parameters: sex (male *versus* female); age groups (≤ 50 *versus* > 50 years); periods of diagnosis (1991–1999 *versus* 2000–2009 *versus* 2010–2021); presentation leucocyte counts (≤ 10 *versus* > 10 × 10^9^/L); presentation platelet counts (≤ 40 *versus* > 40 × 10^9^/L); Sanz risk scores (low *versus* intermediate *versus* high); and treatment regimens (ATRA-based *versus* oral-ATO-based). For 30-day survival, difference between treatment centres (academic *versus* non-academic) was also evaluated. Prognostic factors with P < 0.10 on univariate analysis were further examined by multivariate analyses. Two-tailed P values of < 0.05 were regarded as significant. Statistical analyses were performed with the SPSS version 26.0 (Chicago, IL, USA).

***Epidemiological analyses.*** The age-adjusted annual incidence rates of APL per 100,000 people were calculated using R version 4.0.4 (R Foundation for Statistical Computing, Vienna, Austria), [24] with the World Health Organization (WHO) (2000–2025) standard population and the Hong Kong population as reference. The incidence rate ratios (IRR) of second primary cancers between different age groups, sex, treatment periods and exposure to oral-ATO were obtained with the rateratio() function from the fmsb package within the R software [25]. Hong Kong mid-year population by sex and age from 1991 to 2020 was extracted from The Human Mortality Database (1991–2017) [26] and the Hong Kong Census and Statistics Department (2018–2020) [27]. The World Health Organization standard population data (2000–2025) were downloaded from the Surveillance, Epidemiology and End Result (SEER) website [28].

***Second primary cancers.*** As the number of APL patients was relatively small, the standardized incidence ratio (SIR) was used to determine if the occurrence of second primary cancers was increased in APL. SIR was calculated by indirect standardization (Supplemental file 2).

## Results

***Patient characteristics.*** There were 751 patients (364 males, 387 females) at a median age of 44 (range: 1–97; interquartile range: 31–57) years (Table 1). Patients were treated in three time periods (January 1, 1991–December 31, 1999, N = 146; January 1, 2000–December 31, 2009, N = 257; January 1, 2020–March 31, 2021, N = 348). Conventional high-risk features of platelet ≤ 40 × 10^9^/L and leucocyte > 10 × 10^9^/L were present in 29.4% and 34.5% of cases, with no significant changes during the study periods (Table 1). High-risk Sanz score was present in 34.5% of cases, being comparable in the three time periods (32.3%, 31.3%, 37.7% respectively).

**Table 1:** Clinicopathologic features, treatment and outcome of 751 patients with acute promyelocytic leukaemia in three decades

Features	Total	1990–1999	2000–2009	2010–2021	P value^A^
Sex					
Male	364 (48.5%)	72 (49.3%)	128 (49.8%)	164 (47.1%)	0.788
Female	387 (51.5%)	74 (50.7%)	129 (50.2%)	184 (52.9%)	
Presentation leucocyte ^B^					
≤ 10 × 10^9^/L	481 (65.5%)	90 (67.7%)	176 (68.7%)	215 (62.3%)	0.22
> 10 × 10^9^/L	253 (34.5%)	43 (32.3%)	81 (31.3%)	131 (37.7%)	
Presentation platelet ^B^					
≤ 40 × 10^9^/L	519 (70.6%)	89 (66.9%)	182 (70.8%)	248 (71.9%)	0.56
> 40 × 10^9^/L	216 (29.4%)	44 (33.1%)	75 (29.2%)	97 (28.1%)	
Sanz risk score ^B^					
Low	142 (18.9%)	31 (23.3%)	52 (20.2%)	59 (17.1%)	0.328
Intermediate	340 (45.3%)	59 (44.4%)	125 (48.6%)	156 (45.2%)	
High	253 (34.5%)	43 (32.3%)	81 (31.3%)	131 (37.7%)	
Induction regimen					
ATRA-based	613 (81.6%)	146 (100%)	254 (98.8%)	213 (61.2%)	< 0.001
Oral-ATO-based	138 (18.4%)	0	3 (1.2%)^C^	138 (38.8%)	
CR1 maintenance					
ATRA ± MTX/6-MP	477 (63.5%)	146 (100%)	186 (72.4%)	145 (41.7%)	< 0.001
Oral-ATO/ATRA based	274 (36.5%)	0	71 (27.6%)	203 (58.3%)	
First relapse	97/607 (16%)	26/107(24.3%)	55/203 (27.1%)	16/297(5.4%)	< 0.001
HSCT					
Autologous	13 (1.7%)	6 (4.1%)	5 (1.9%)	2 (0.6%)	
Allogeneic	10 (1.3%)	9 (6.2%)	1 (0.4%)	0	
Second cancers	21 (2.8%)	8 (5.5%)	12 (4.7%)	1 (0.3%)	
Deaths					
All causes	271	75 (51.4%)	109 (42.4%)	87 (25%)	< 0.001
Early deaths	144 (53.1%)	39 (52%)	54 (49.5%)	51 (58.6%)	< 0.001
Vascular events	7 (2.6%)	0	3 (2.8%)	4 (4.6%)	
Sepsis	18 (6.6%)	3 (4%)	11 (10.1%)	4 (4.6%)	
Second cancers	12 (4.4%)	4 (5.3%)	8 (7.3%)	0	
HSCT-related	7 (2.6%)	7 (9.3%)	0	0	
Refractory leukaemia	49 (18.1%)	19 (25.3%)	26 (23.9%)	4 (4.6%)	
Others	6 (2.2%)	2 (2.7%)	1 (0.9%)	3 (3.4%)	
Not available	28 (10.3%)	1 (1.3%)	6 (5.5%)	21 (24.1%)	

ATRA: all trans retinoic acid; ATO: arsenic trioxide; CR1: first complete remission; MTX: methotrexate; 6-MP: 6-mercaptopurine; HSCT: haematopoietic stem cell transplantation;

A: statistical evaluation of parameters in the three time periods was performed with Chi Square test

B: Seventeen patients (2.3%) had missing presentation leucocyte counts and sixteen patients (2.1%) had missing presentation platelet counts, so that the Sanz risk scores could not be determined for these cases

C: These three patients were unfit for chemotherapy and hence were treated with oral-ATO during this period when ATRA-based chemotherapy was the standard

**Fig. 1:**
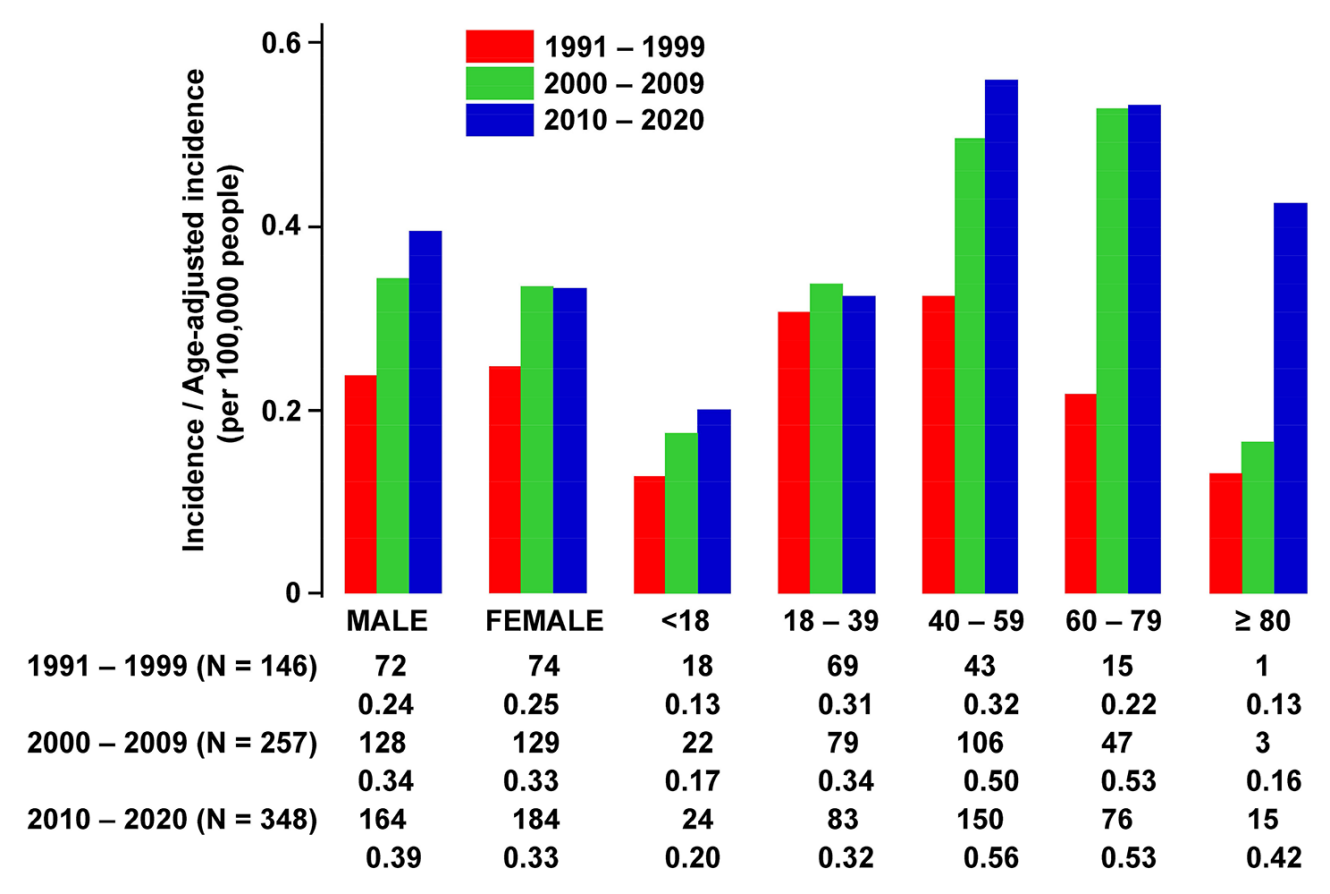
Annual incidences / Age-adjusted annual incidences of acute promyelocytic leukaemia over a 30-year period. The Y axis represented annual incidences for males and females, and age-adjusted annual incidences for different age groups. The actual numbers of patients and the annual incidences / age-adjusted annual incidences for each time period were given under the different analysed groups. Annual incidence was studied until end of 2020, because only three months had elapsed for the year 2021, which was not included in the analysis

***Epidemiological changes.*** There was a progressive increase in annual incidence rates (per 100,000 people) with time (0.24 in 1991–1999, 0.34 in 2000–2009, 0.36 in 2010–2021) (Fig. 1), giving an overall incidence of 0.32 (see Supplemental file 3 A for incidence in each calendar year). In the three time periods, two changes were observed. Firstly, there was a gradual shift of occurrence of APL to the older age groups. In 1991–1999, the highest age-adjusted incidences occurred in the groups 18–39 years and 40–59 years. However, in 2000–2009 and 2010–2020, the highest age-adjusted incidences had shifted to the groups 40–59 years and 60–79 years. Secondly, the age-adjusted annual incidences also increased with time. The increase was only very modest in the groups < 18 years and 18–39 years, but was more obvious in the groups 40–59 years (0.32 in 1991–1999; 0.50 in 2000–2009; 0.56 in 2010–2021) and 60–79 years (0.22 in 1991–1999; 0.53 in 2000–2009; 0.53 in 2010–2021). The most notable increase was in the group > 80 years (0.13 in 1991–1999; 0.26 in 2000–2009; 0.42 in 2010–2021).

***Treatment outcome.*** For induction, all patients received ATRA-based regimens during 1991–1999 and 2000–2009 (except three patients unfit for chemotherapy who received oral-ATO-based regimens); with significantly more patients (38.8%) receiving oral-ATO-based regimens from 2010 to 2021 (P < 0.001) (Table 1). For CR1 maintenance, all cases received ATRA-based regimens during 1991–1999, whereas afterwards significantly more patients received oral-ATO-based regimens, increasing from 27.6% in 2000–2009 to 58.3% in 2010–2021 (P < 0.001). The increasing use of oral-ATO-based regimens in induction and CR1 maintenance significantly decreased the incidence of R1 from 24.3% in 1990–1999 and 27.1% in 2000–2009 to merely 5.4% in 2010–2021 (P < 0.001) (Table 1).

**Fig. 2:**
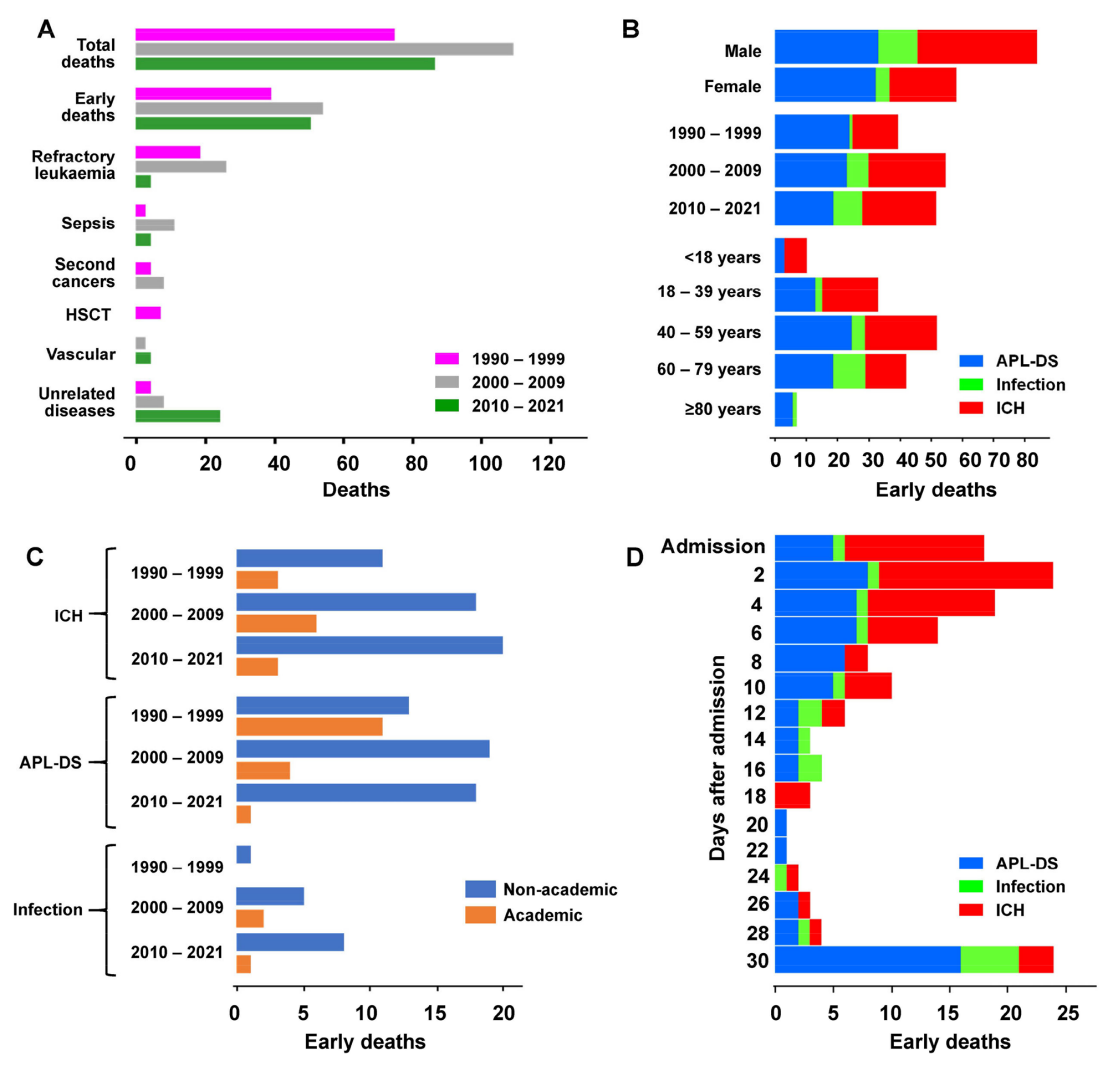
Mortalities of patients with acute promyelocytic leukaemia A. Total number of deaths due to different causes according to the time period studied B. Early deaths and their respective proportions due to acute promyelocytic leukaemia differentiation syndrome (APL-DS), infections and intracerebral haemorrhage (ICH) of the various parameters studied C. Early deaths due to ICH, APL-DS and infection in the different time periods studied, according to treatment in academic or non-academic centres D. Early deaths due to ICH, APL-DS and infections during the first 30 days of presentation

***Mortalities.*** After a median follow-up of 75 (interquartile range: 14–161) months, there were 271 deaths (36.1%) (Fig. 2A). The two most important causes were ED and refractory leukaemia (Supplemental file 3B). In the three time periods, there was no improvement in ED (1991–1999, N = 39; 2000–2009: N = 54; 2010–2021, N = 51) (Fig. 2A). However, there was a significant decrease in death due to refractory leukaemia (1991–1999, N = 19; 2000–2009: N = 26; 2010–2021, N = 4) (Fig. 2A). Hence, in 2010–2021, with the decrease in refractory leukaemia and increase in older patients, systemic diseases unrelated to APL became the second most frequent cause of death after ED.

***ED.*** During the study period, ED occurred in 144 patients (19.2% of all patients), accounting for 53% of all deaths. Remarkably, EDs happened almost exclusively in cases receiving ATRA-based induction (139/144, 96.5%), resulting in a 30-day mortality of 22.7% (139/613) in these patients. On the contrary, only 3.5% (5/144) of EDs happened in patients receiving oral-ATO-based induction, resulting in a 30-day mortality of only 3.6% (5/138). The three most important causes of ED were APL differentiation syndrome (APL-DS; N = 66; with 61 due to pulmonary complications and 5 due to renal failure), intracerebral haemorrhage (ICH; N = 61) and infection (N = 11). EDs were more frequent in men, owing mainly to increased ICH (Fig. 2B). Although ED due to APL-DS decreased during the study periods, ED due to ICH did not improve. The proportion of EDs due to APL-DS and ICH remained similar in different age groups (Fig. 2B). However, EDs due to ICH, APL-DS and infections differed according to whether the treatment centres were academic (Queen Mary Hospital) or non-academic (all other hospitals) (Fig. 2C). In the three study periods, ICH were 3–6 times more frequent in non-academic centres. For APL-DS, mortalities in 1991–1999 were comparable between academic and non-academic centres. In 2000–2009 and 2010–2021, mortality due to APL-DS continued to drop in the academic centre, but remained high in non-academic centres. Similarly, mortalities due to infection were also fewer in the academic centre during the study periods. In the first week post-admission, ICH was the predominant cause of death, followed by APL-DS (Fig. 2D). APL-DS became the predominant cause of death towards the end of the first 30 days.

**Fig. 3:**
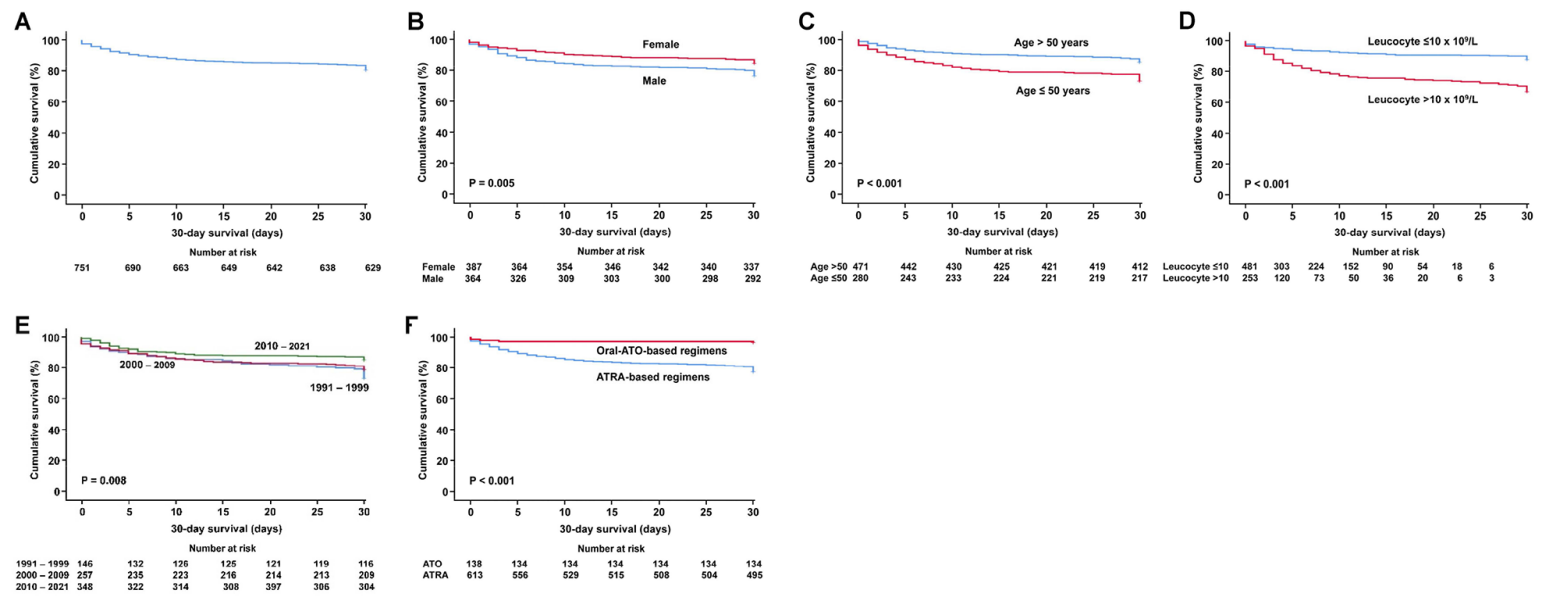
30-day survivals in newly-diagnosed APL patients. Only prognostic parameters with statistical significance on multivariate analyses were shown

***30-day survival.*** The 30-day survival was 80.8% (Fig. 3A). On univariate analysis, 30-day survival was inferior with male sex (P = 0.005), age > 50 years (P < 0.001), leucocyte > 10 × 10^9^/L (P < 0.001), diagnosis in 1991–1999 and 2000–2009 (P = 0.008) (Fig. 3B–E), high-risk Sanz score (P < 0.001) (Supplemental file 4); and superior with the use of oral-ATO-based induction regimens (P < 0.001) (Fig. 3F). On multivariate analysis, 30-day survival remained significantly inferior with male sex, age > 50 years, leucocyte > 10 × 10^9^/L, diagnosis in 1991–1999 and 2000–2009; and superior with use of oral-ATO-based induction regimens (Table 2).

**Table 2:** Significant prognostic factors for survivals in a cohort of newly-diagnosed patients with acute promyelocytic leukaemia

		Univariate analysis						Multivariate analysis				
Parameters	Number	HR	95% C.I.	P value				H.R.	95% C.I.	P value		
30-day survival (N = 751)												
Sex												
Male	364	1.60	1.15–2.24	0.005				1.48	1.05–2.09	0.03		
Female	387	0.62	0.45–0.87					0.68	0.48–0.95			
Age												
≤ 50 years	471	0.51	0.37–0.70	< 0.001				0.37	0.26–0.53	< 0.001		
> 50 years	280	1.96	1.42–2.72					2.71	1.91–3.86			
Period of diagnosis												
1991–1999	146	1.90	1.25–2.89	0.008				1.96	1.23–3.12	0.02		
2000–2009	257	1.49	1.02–2.18					1.27	0.85–1.89			
2010–2021	348	0.53	0.35–0.80					0.51	0.32–0.81			
Leucocyte count												
≤ 10 × 10^9^/L	481	0.33	0.23–0.46	< 0.001				0.32	0.23–0.45	< 0.001		
> 10 × 10^9^/L	253	3.09	2.20–4.32					3.14	2.23–4.43			
Induction regimens												
ATRA-based	613	6.86	2.81–16.73	< 0.001				6.14	2.44–15.43	< 0.001		
Oral-ATO-based	138	0.15	0.06–0.36					0.16	0.07–0.41			
**Overall survival (N = 751)**												
Sex												
Male	364	1.47	1.16–1.89	0.001				1.35	1.06–1.72	0.015		
Female	387	0.68	0.53–0.86					0.74	0.58–0.94			
Age												
≤ 50 years	471	0.49	0.39–0.62	< 0.001				0.35	0.27–0.45	< 0.001		
> 50 years	280	2.04	1.61–2.60					2.88	2.23–3.73			
Leucocyte count												
≤ 10 × 10^9^/L	481	0.51	0.40–0.65	< 0.001				0.51	0.39–0.65	< 0.001		
> 10 × 10^9^/L	253	1.98	1.55–2.52					1.98	1.54–2.54			
Sanz score												
Low-risk	142	0.66	0.47–0.91	< 0.001				1.59	1.12–2.24	0.01		
Intermediate-risk	340	0.44	0.33–0.57					0.63	0.45–0.89			
High-risk	253	1.53	1.10–2.11					1.98	1.54–2.54			
Treatment regimens												
ATRA-based	469	5.26	3.57–7.69	< 0.001				5.56	3.84–8.33	< 0.001		
Oral-ATO-based	282	0.19	0.13–0.28					0.18	0.12–0.26			
**Post-30-day survival (N = 607)**												
Age												
≤ 50 years	402	0.45	0.31–0.64	< 0.001				0.29	0.20–0.43	< 0.001		
> 50 years	205	2.23	1.57–3.17					3.40	2.33–4.98			
Treatment regimens												
ATRA-based	331	2.94	1.92–4.55	< 0.001				3.03	1.82–5.26	< 0.001		
Oral-ATO-based	276	0.34	0.22–0.52					0.33	0.19–0.55			
**Relapse free survival (N = 607)**												
Period of diagnosis												
1991–1999	107	4.38	2.34–8.16	< 0.001				2.51	1.27–4.99	< 0.001		
2000–2009	203	4.52	2.58–7.90					3.40	1.90–6.09			
2010–2021	297	0.23	0.12–0.43					0.40	0.25–0.73			
Treatment regimens												
ATRA-based	331	3.45	2.13–5.56	< 0.001				2.33	1.37–4.00	0.002		
Oral-ATO-based	276	0.29	0.18–0.47					0.43	0.25–0.73			

h: hazard ratio; C.I.: confidence interval; ATRA: all trans retinoic acid; ATO: arsenic trioxide

**Fig. 4:**
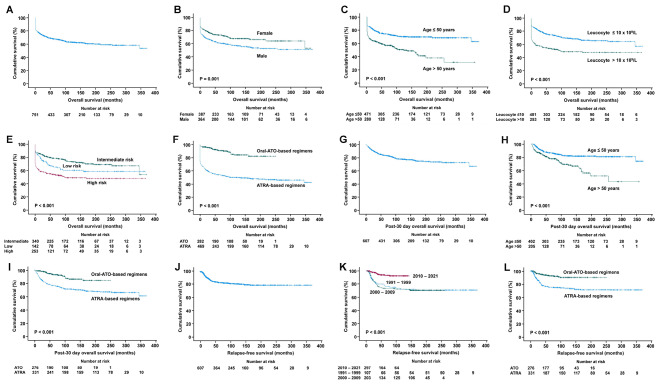
Kaplan Meier survival curves of the entire cohort of patients with acute promyelocytic leukaemia. Only prognostic parameters with statistical significance on multivariate analyses were shown

***OS of the entire cohort.*** The 5-year and 10-year OS were 68.1% and 63.3% (Fig. 4A). On univariate analysis, OS was inferior with male sex (P = 0.001), age > 50 years (P < 0.001), leucocyte > 10 × 10^9^/L (P < 0.001), high-risk Sanz score (P < 0.001) (Fig. 4B–E); and superior with the use of oral-ATO-based induction/maintenance regimens (P < 0.001) (Fig. 4F) and diagnosis in 2010–2021 (P < 0.001) (Supplemental file 5). On multivariate analysis, OS remained significantly inferior with male sex, age > 50 years, leucocyte > 10 × 10^9^/L, high-risk Sanz score; and superior with oral-ATO-based induction/maintenance regimens (Table 2).

***Post-30-day OS of the entire cohort.*** The 5-year and 10-year post-30-day OS were 84.0% and 78.1% (Fig. 4G). On univariate analysis, post-30-day OS was inferior with age > 50 years (P < 0.001) (Fig. 4H), platelet > 40 × 10^9^/L (P = 0.003) (Supplemental file 5); and superior with intermediate Sanz score (P = 0.005) (supplemental file 5), diagnosis in 2010–2021 (P = 0.023) (Supplemental file 5), and oral-ATO-based induction/maintenance regimens (P < 0.001) (Fig. 4I). On multivariate analysis, post-30-day survival remained significantly inferior with age > 50 years and superior with oral-ATO-based induction/maintenance regimens (Table 2).

***RFS of the entire cohort.*** In 607 CR1 patients, 97 patients (16%) relapsed after a median of 70 (interquartile range: 27–155) months. The 5-year RFS was 83.8% (Fig. 4J). On univariate analysis, RFS was significantly superior with age > 50 years (P = 0.031) (Supplemental file 5), diagnosis in 2010–2021 (P < 0.001), and the use of oral-ATO-based induction/maintenance regimens (P < 0.001) (Fig. 4K,L). On multivariate analysis, RFS remained significantly superior with diagnosis in 2010–2021 and use of oral-ATO-based induction/maintenance regimens (Table 2).

**Fig. 5:**
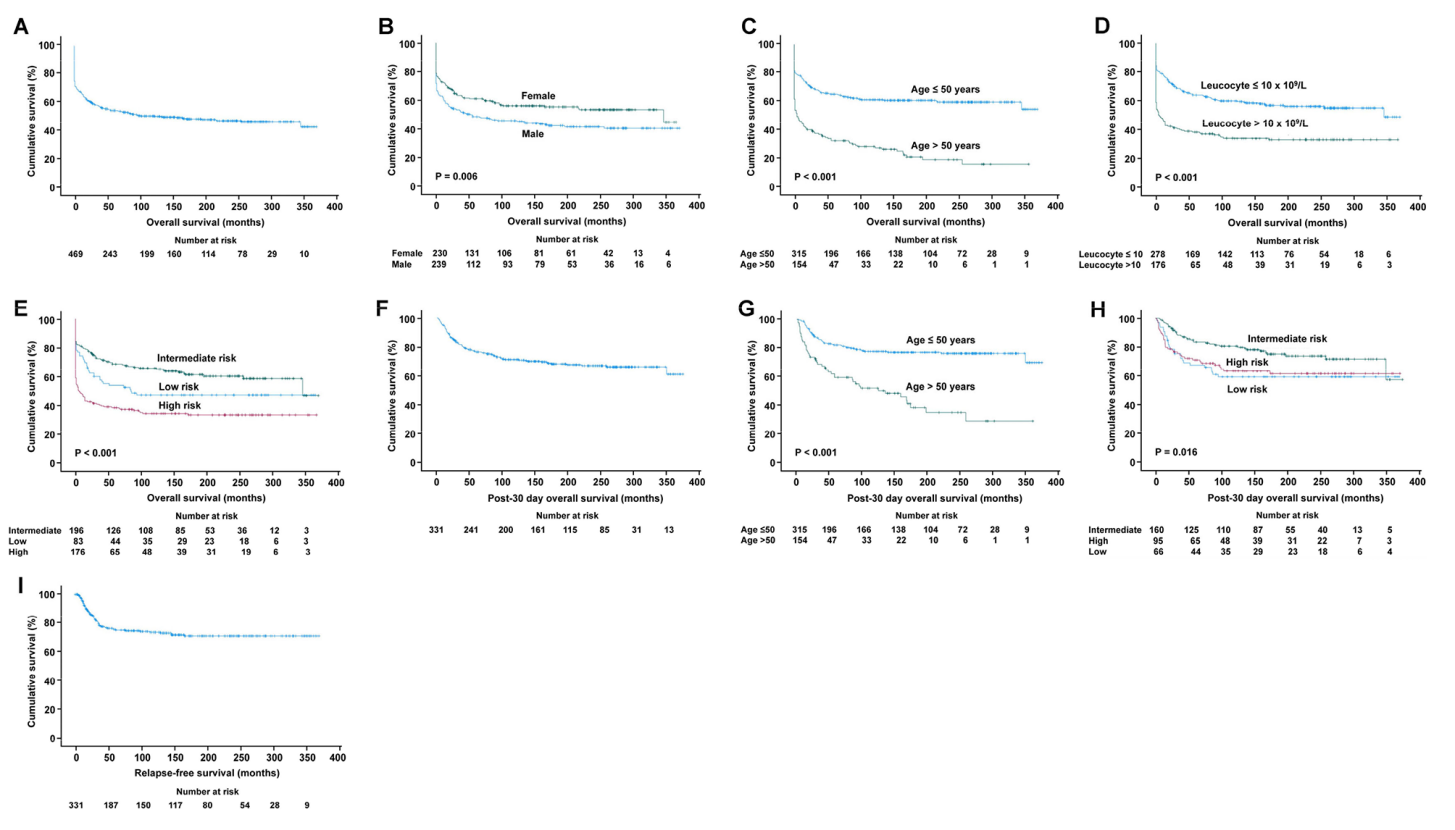
Kaplan Meier survival curves of patients with acute promyelocytic leukaemia treated with all-trans retinoic acid based regimens. Only prognostic parameters with statistical significance on multivariate analyses were shown

***Survivals and prognostic factors in the ATRA-based cohort.*** In 469 patients receiving ATRA-based regimens without exposure to oral-ATO (ATRA-based cohort), the 5-year and 10-year OS were 54.5% and 50.5% (Fig. 5A). On both univariate and multivariate analyses, OS was significantly inferior with male sex (P = 0.006), age > 50 years (P < 0.001), leucocyte > 10 × 10^9^/L (P < 0.001) and high-risk Sanz score (P < 0.001) (Fig. 5B–E) (Table 3). The 5-year and 10-year post-30-day OS were 76.5% and 71.3% (Fig. 5F). On univariate analysis, post-30-day OS was inferior with age > 50 years (P < 0.001) (Fig. 5G), platelet > 40 × 10^9^/L (P = 0.01) (Supplemental file 6); and superior with intermediate-risk Sanz score (P = 0.016) (Fig. 5H). On multivariate analysis, post-30-day OS remained significantly inferior with age > 50 years and superior with intermediate Sanz score (Table 3). The 5-year RFS in this cohort was 76.5% (Fig. 5I). No parameters impacted significantly on RFS (Supplemental file 6).

**Table 3:** Significant prognostic factors for survivals in newly-diagnosed patients with acute promyelocytic leukaemia treated with ATRA-based and oral-ATO-based regimens

			Univariate analysis									Multivariate analysis				
Parameters	Number		HR	95% C.I.				P value				H.R.	95% C.I.	P value		
**ATRA-based regimens**																
**Overall survival (N = 469)**																
Sex																
Male	239		1.39	1.08–1.79				0.01				1.41	1.08–1.82	0.012		
Female	230		0.72	0.56–0.93								0.71	0.55–0.93			
Age																
≤ 50 years	315		0.38	0.29–0.49				< 0.001				0.36	0.27–0.46	< 0.001		
> 50 years	154		2.62	2.03–3.39								2.81	2.16–3.66			
Leucocyte count																
≤ 10 × 10^9^/L	278		0.50	0.39–0.65				< 0.001				0.42	0.31–0.57	< 0.001		
> 10 × 10^9^/L	176		2.00	1.54–2.59								1.53	1.07–2.18			
Sanz score																
Low-risk	83		0.65	0.46–0.92				< 0.001				0.65	0.46–9.30	< 0.001		
Intermediate-risk	196		0.44	0.33–0.59								0.41	0.31–0.56			
High-risk	176		1.55	1.09–2.20								1.53	1.08–2.18			
**Post-30-day survival (N = 331)**																
Age																
≤ 50 years	247		0.31	0.21–0.45				< 0.001				0.26	0.17–0.39	< 0.001		
> 50 years	84		3.26	2.20–4.83								3.90	2.59–5.89			
Sanz score																
Low-risk	83		1.06	0.64–1.17				0.02				0.92	0.55–1.54	0.001		
Intermediate-risk	196		0.57	0.36–0.91								0.45	0.28–0.72			
High-risk	176		0.94	0.57–1.57								1.09	0.65–1.82			
**Oral-ATO-based regimens**																
**Overall survival (N = 282)**																
Age																
≤ 50 years	156		0.44	0.21–0.92				0.03				-*	-	-		
> 50 years	256		3.26	1.09–4.66								-	-			

HR: hazard ratio; C.I.: confidence interval; ATRA: all trans retinoic acid; ATO: arsenic trioxide;*: only factor significant on univariate analysis, hence multivariate analysis not performed

**Fig. 6:**
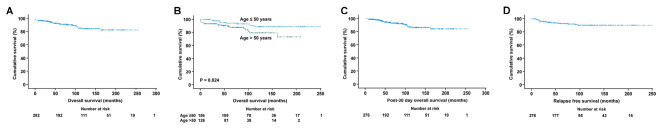
Kaplan Meier survival curves of patients with acute promyelocytic leukaemia treated with oral arsenic trioxide based regimens. Only prognostic parameters with statistical significance on multivariate analyses were shown

***Prognostic indicators in the oral-ATO-based cohort.*** In 282 patients receiving oral-ATO-based induction/maintenance (oral-ATO-based cohort), the 5-year and 10-year OS were 91.5% and 84.6% (Fig. 6A). On univariate analysis, only age > 50 years was associated with inferior OS (P = 0.03) (Table 3) (Fig. 6B). The 5-year and 10-year post-30-day OS were 93.5% and 86.4% (Fig. 6C); and the 5-year RFS was 93.3% (Fig. 6D). No factors significantly impacted on post-30-day OS and RFS (Supplemental file 7).

***Second primary cancers.*** In 607 CR1 patients, 21 patients (6 males, 15 females) developed second cancers (breast cancer, N = 5; colorectal cancer, N = 3; endometrial cancer; N = 2; myelodysplastic syndrome, N = 2; esophageal cancer, N = 1; thyroid cancer, N = 1; nasopharyngeal cancer, N = 1; parotid cancer, N = 1; transitional carcinoma of the ureters, N = 1; lung cancer, N = 1; renal cell carcinoma; cholangiocarcinoma, N = 1; tongue cancer, N = 1) at a median of 8 years (range: 1–24 years) after the diagnosis of APL (Supplemental file 8). The incidence was highest in females (N = 15) (SIR: 4.54, 95% confidence interval, C.I.: 2.66–7.55), and in patients diagnosed at the age of 40–59 years (N = 12) (SIR: 5.53, 95% confidence interval C.I.: 3.02–9.69), during 1991–1999 (N = 8) (SIR: 5.94, 95% C.I.: 2.79–11.75) and during 2000–2009 (N = 12) (SIR: 5.21, 95% C.I.: 2.85–9.16). Thirteen patients were oral-ATO-exposed (oral-ATO maintenance, N = 8; oral-ATO for re-induction of CR2, N = 3; autologous HSCT for oral-ATO-induced CR2 or beyond, N = 2); whereas eight patients were oral-ATO-naïve. There was no significant difference in second cancers in patients exposed or naïve to oral-ATO (IRR: 2.14; 95% C.I.: 0.89–5.17) (Supplemental file 9). However, there was an overall significant increase in second cancers in APL patients as compared to the general population (IRR: 14.3, P < 0.001), which applied to both sexes, whether or not oral-ATO had been used, age groups 18–39 years and 40–59 years, and treatment during 1991–1999 and 2000–2009 (Supplemental file 10). In patients of < 18 years, 60–75 years and > 80 years and those treated during 2010–2020, second cancers were not increased.

## Discussion

We observed a progressive increase in the incidence (per 100,000 people) of APL over the past three decades, with a 50% increase from 0.24 in 1991–2000 to 0.36 in 2010–2021. Furthermore, we found a shift of the peak age incidence from 18–39/40–59 years in 1991–2000 to 40–59/60–79 years in 2000–2009 and 2010–2021. Finally, from 1991 to 2000 to 2010–2021, there was a > 3-fold increase in age-adjusted incidence in patients > 80 years. Although epidemiologic studies of APL are limited, such trends were also observed in three SEER-based studies covering the periods 1992–2001, 1975–2008 and 2000–2014 [17, 18, 29, 30]. In these studies, the mean annual incidences increased from 0.20 (in 1992–2001) to 0.27 (in 2000–2008) and to 0.31 (in 2000–2014) [17, 18, 29, 30]. All studies observed increasing incidences with age, the highest incidence observed in people aged ≥ 60–65 years. This was different from much earlier studies, where the incidence of APL was regarded to be stable with age, [30, 31] leading to the hypothesis of a single rate-limiting mutation to explain the apparent constant incidence [31]. On the contrary, our observations and those of others support a more conventional multi-hit model, with accumulation and interaction of mutations throughout life, resulting in rising incidences with age. Biologically, mice transgenic for the fusion gene *PML*::*RARA* manifested a pre-leukaemic state of deranged myeloid maturation, with an APL-like leukaemia only developing if collaborating mutations such as internal tandem duplication of *FLT3* were present [32–34]. Hence, although doubtlessly *PML*::*RARA* fusion is the key driver event, APL might still evolve through a multistep process of leukaemogenesis.

In contrast to other types of acute myeloid leukaemia (AML) where the male:female incidence rate is about 1.5:1, we observed a similar incidence rate of APL in men (0.33) and women (0.32). The relatively higher frequency of APL in women has also been reported in population-based studies and clinical trials [30]. It has been postulated that estrogen, which belongs to the same superfamily as retinoid receptors, might act synergistically to affect the binding of the aberrant PML/RARA protein to the RXR receptor [30].

In this study, EDs accounted for more than half of the total mortalities. In multicenter clinical trials of patients treated with ATRA, ATO and anthracyclines, early death rates of only 3–10% were reported [7, 8, 35–37]. However, population-based studies in unselected patients reported otherwise, with EDs varying from 9.6–61.5% [17, 18, 38–43]. Our ED rate of 19.2% was within this range. Efforts in the development of international recommendations have led to a gradual improvement of ED, falling from 28% in the 1990s to approximately 15% in the past two decades [17, 44, 45]. Reported risks for EDs included older age, high-risk disease, poor performance, co-existing infections; [46] and factors that increased fatal haemorrhages, including high leucocyte count, elevated lactate dehydrogenase, low fibrinogen, impaired coagulation parameters and APL-DS [47–51]. Delays in ATRA administration contributed significantly to EDs, especially at the community care level where ATRA might not be immediately available [51–53]. In addition to confirming some of these observations, there were two additional findings in this study. Firstly, use of oral-ATO-based inductions almost completely abrogated EDs. Secondly, EDs did not improve in non-academic centres, but progressively decreased in the academic centre, mainly related to reduction of mortality due to APL-DS.

The impact of EDs on survivals was further shown in the analysis of prognostic factors. Male sex, age > 50 years, leucocyte count > 10 × 10^9^/L and use of ATRA-based instead of ATO-based induction regimens significantly increased EDs. These negative prognostic factors were precisely those that led to significantly inferior OS for the entire cohort. When EDs were factored out, survivals of 68.1% and 63.3% (OS) were improved to 84% and 78.1% (post-30-day OS) at 5 and 10 years.

Refractory leukaemia was the other main cause of mortality in this study. However, with the increasing use of oral-ATO-based regimens, refractory leukaemia was largely prevented during 2010–2020. Consequently, post-30-day OS was significantly improved with oral-ATO-based regimens, and RFS was significantly improved with oral-ATO-based regimens and diagnosis in 2010–2020, when oral-ATO-based regimens were predominantly used.

In the above analyses, use of oral-ATO-based regimens, resulted in significantly superior results in all survivals (30-day survival, OS, post-30-day OS and RFS) by decreasing EDs and refractory leukaemia. To fully evaluate the importance of treatment, we separately analysed our patients according to treatment with ATRA-based and oral-ATO-based regimens. ATRA-based regimens gave 5-year and 10-year OS of merely 54.5% and 50.5%; 5-year and 10-year post-30-day OS of 76.5% and 71.3%, and 5-year RFS of 76.5%. Conventional prognostic factors, including male sex, age > 50 years and Sanz score, expectedly portended inferior survivals. On the other hand, oral-ATO-based regimens gave much better survivals. The 5-year and 10-year OS were 91.5% and 84.6%, comparable with the 5-year and 10-year post-30-day OS of 93.5% and 86.4%; reflecting the importance of reduction of EDs on OS. The 5-year RFS was also excellent at 93.3%, due to significantly decreased relapses. With oral-ATO-based regimens, conventional prognostic indicators (leucocyte count, platelet count, Sanz score) were no longer relevant. The only factor negatively impacting on OS was age > 50 years, reflecting deaths from non-leukaemic age-related diseases.

With APL potentially curable, risks of second primary cancers have become pertinent. In a recent population-based study in the United States, the absolute incidence of second cancers per 1,000 person-months was increased from 1.4 in i.v.-ATO-naïve patients to 3.4 in i.v.-ATO-exposed patients [21]. However, in another population-based study, second cancers in i.v.-ATO-exposed patients were not significantly increased compared with the general population [17]. In APL patients treated with ATRA ± anthracycline, there were increased risks of developing liver cancers, salivary gland cancers and soft tissue malignancies compared with other subtypes of AML, [20] suggesting a potentially carcinogenic role of ATRA. In our study, we observed an increased risk of second cancers in APL patients compared to the general population. The increased risk was not associated with the use of oral-ATO, implying that chemotherapy might be involved. In subgroup analysis, second cancers were not increased in patients aged < 18 years and > 60 years; probably reflecting the low and high risks of cancers in any case in these age groups. Interestingly, second cancers were also not increased in patients treated during 2010–2018, a period when oral-ATO-based regimens with omission of daunorubicin were increasingly used. Whether the use of a chemotherapy-free strategy in APL [8] may reduce second cancers warrants further investigations. In addition, this population-based study on the epidemiology and outcome was entirely based in Hong Kong and its applicability in other patient populations such as Hispanics, Caucasians and Africans has to be carefully examined.

## Conclusion

Results of our study have important implications on how to further improve the service to APL patients. EDs account for the majority of deaths and are clearly the biggest hurdle. Education of frontline healthcare workers in diagnosing APL and vigorously treating the associated coagulopathy is key to preventing ICH. It is imperative that ATRA should be available at the community care level [53]. Referral to academic centres is advantageous. Otherwise, early recognition of APL-DS leading to its timely treatment should be inculcated in physicians at non-academic centres. Oral-ATO-based regimens significantly improved all survivals, so that ATO (i.v. or oral) should be incorporated into all phases of treatment. With the gradual shift of APL to older patients, more resources and attention should be devoted to the treatment of other comorbidities to reduce non-leukaemic mortalities. In this respect, the use of an entirely non-chemotherapy approach in elderly patients to avoid toxicities should be explored. Finally, with APL now largely curable, decreasing the risks of second primary cancers by reducing or omitting chemotherapy should be a therapeutic target.

## Electronic supplementary material

Below is the link to the electronic supplementary material.

Supplementary Material 1

Supplementary Material 2

Supplementary Material 3

Supplementary Material 4

Supplementary Material 5

Supplementary Material 6

Supplementary Material 7

Supplementary Material 8

Supplementary Material 9

Supplementary Material 10

Supplementary Material 11

## Data Availability

The datasets used and/or analysed during the current study are available from the corresponding author on reasonable request.
