# Supplementary material for: Acute promyelocytic leukaemia: population-based study of epidemiology and outcome with ATRA and oral-ATO from 1991 to 2021
Source: BMC Cancer. 2023 Feb 10;23:141. doi: 10.1186/s12885-023-10612-z (PMC9921648; doi:10.1186/s12885-023-10612-z)
Supplement: Supplementary file 1 — Supplementary Material 1 [file 12885_2023_10612_MOESM1_ESM.pdf]

Supplemental file 1. Treatment regimens for acute promyelocytic leukemia in Hong Kong.

1991-2021
Age 18-69 without cardiac comorbidities

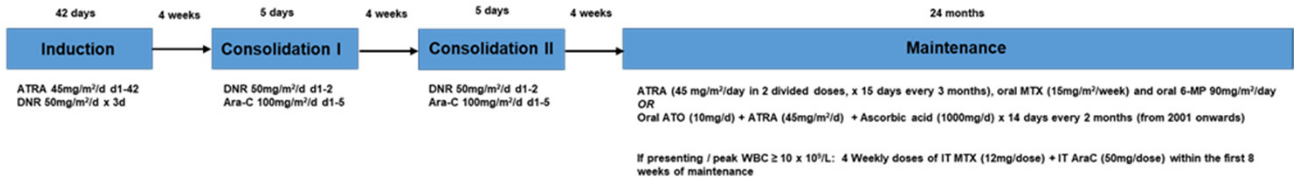

1991-2021
Age ≥ 70 or with severe cardiac co-morbidities or ECOG ≥ 2

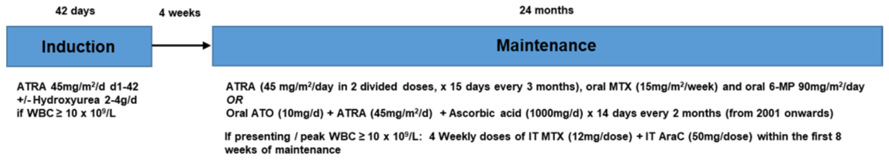

2013-2017 – APL001 study
Age 18-69 without cardiac comorbidities

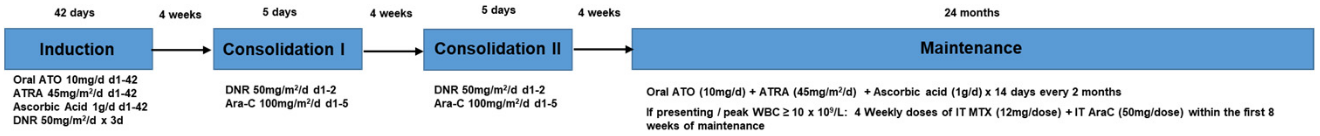

2013-2017 APL001 study
Age ≥ 70 or with severe cardiac comorbidities or ECOG ≥ 2

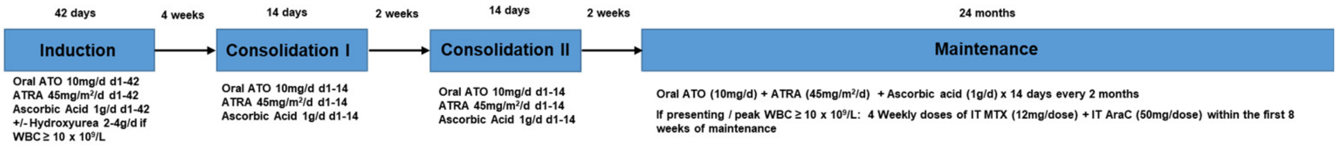

APL002 study 2018-2021
Age ≥ 18 and presenting WBC < 10 x 10⁹/L or Age ≥ 65 regardless of WBC

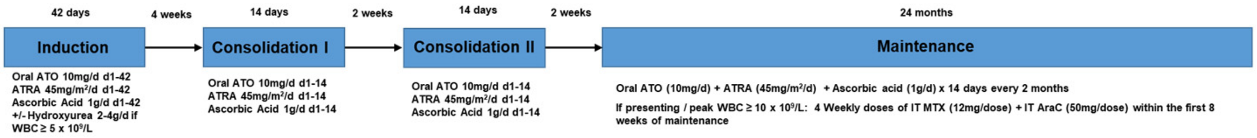

Age ≥ 18 and presenting WBC ≥ 10 x 10⁹/L without cardiac comorbidities

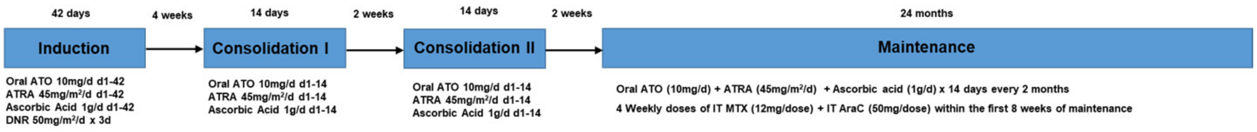

2007-2020 ICC APL Study 01
Age < 18 years

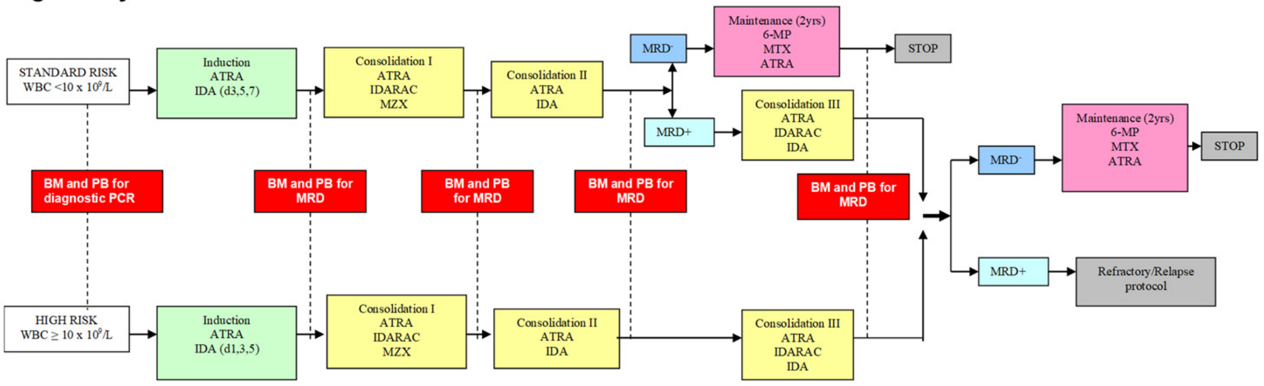

ATRA: all-*trans*-retinoic acid; DNR: daunorubicin; AraC: cytarabine; ATO: arsenic trioxide; MTX: methotrexate; 6-MP: 6-mercaptopurine; IT: intrathecal; WBC: white blood cell; IDA: idarubicin; IDARAC: intermediate dose cytarabine; MZX: mitoxantrone; BM: bone marrow; PB: peripheral blood; MRD: measurable residual disease; d: day/days; x: for; +: plus/positive
