# Supplementary material for: Acute promyelocytic leukaemia: population-based study of epidemiology and outcome with ATRA and oral-ATO from 1991 to 2021
Source: BMC Cancer. 2023 Feb 10;23:141. doi: 10.1186/s12885-023-10612-z (PMC9921648; doi:10.1186/s12885-023-10612-z)
Supplement: Supplementary file 2 — Supplementary Material 2 [file 12885_2023_10612_MOESM2_ESM.pdf]

Supplemental file 2. Calculation of standardized incidence ratio (SIR) of second primary cancers in APL patients.

The SIR of second cancer for APL patients was calculated by dividing the observed number of second cancers by the expected number of APL patients in the Hong Kong population without other primary cancers.

Expected number of APL patients was obtained as follows:

Expected number of APL patients:

$$\sum\ (\,crude\ rate_{ij}\ \times\ standard\ population_{ij}) =$$

$$=\sum\ (\frac{Observed\ number\ of\ APL\ patients_{ij}}{Population_{ij}} \times\ standard\ population_{ij})$$

where
$crude\ rate_{ij}$ = The crude rate of APL patients in the entire Hong Kong population by age and sex, which was obtained by dividing

$Observed\ number\ of\ APL\ patients_{ij}$ (Observed number of APL patients in Hong Kong by age and sex) by

$Population_{ij}$ (Hong Kong mid-year population by age and sex)

$standard\ population_{ij}$ was the standardized population following the WHO (2000-2025) standard population data by age and sex
