# Supplementary figures and images for: Acute promyelocytic leukaemia: population-based study of epidemiology and outcome with ATRA and oral-ATO from 1991 to 2021
Source: BMC Cancer. 2023 Feb 10;23:141. doi: 10.1186/s12885-023-10612-z (PMC9921648; doi:10.1186/s12885-023-10612-z)

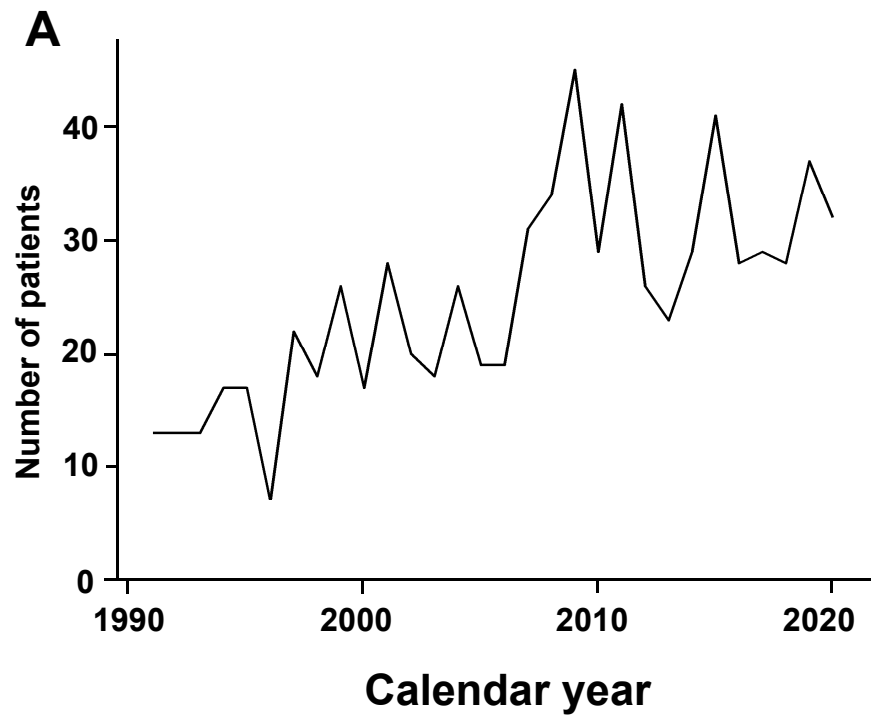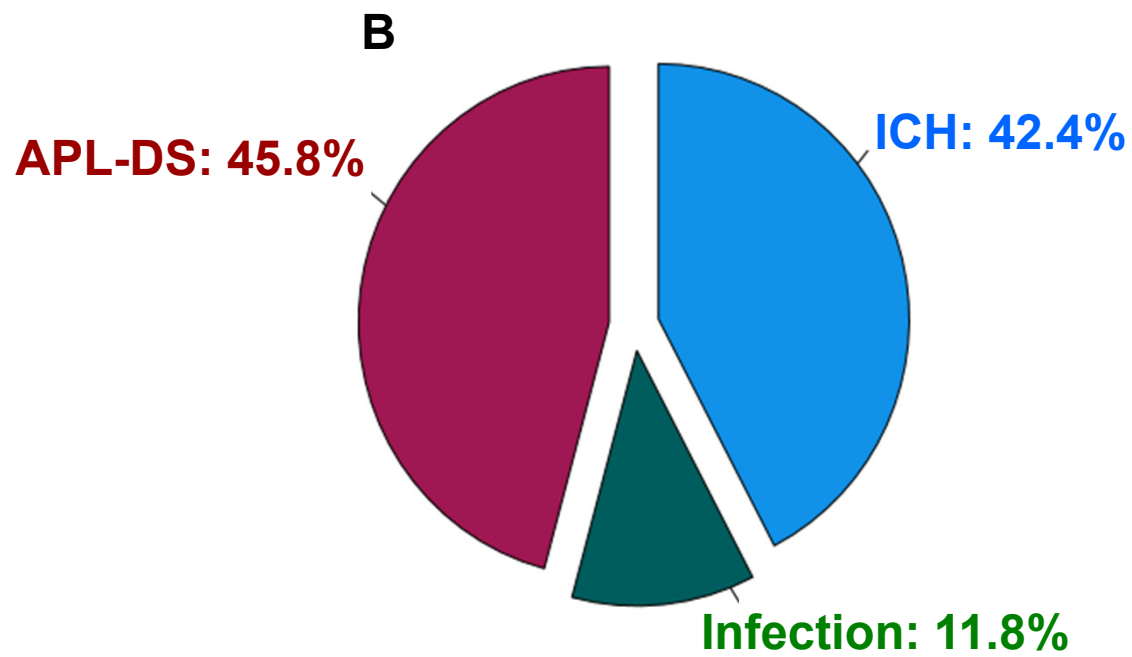

Supplement: Supplementary file 3 — Supplementary Material 3 [file 12885_2023_10612_MOESM3_ESM.pdf]

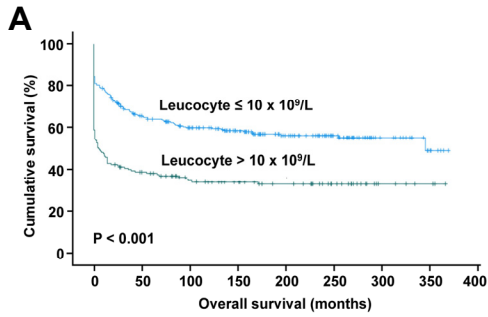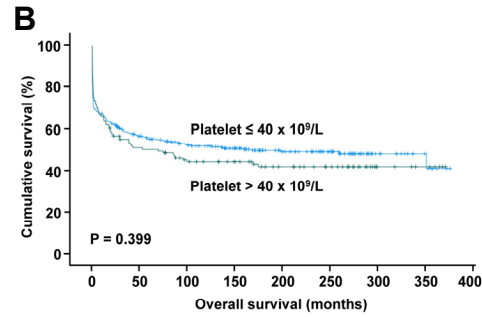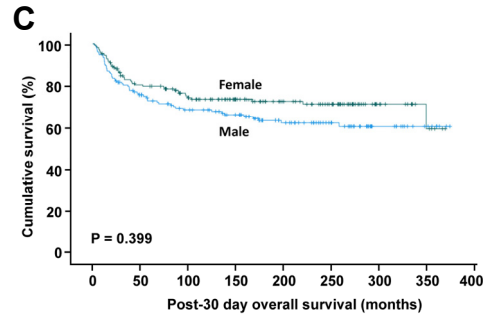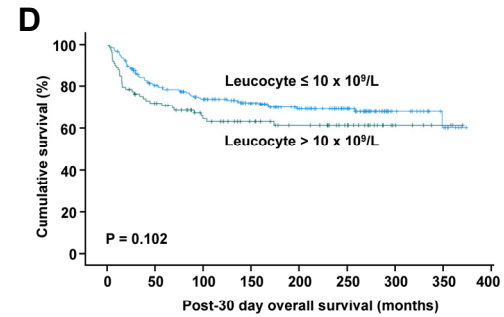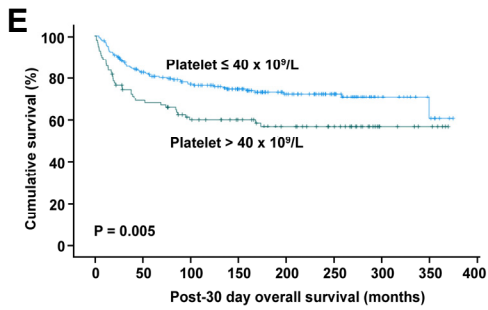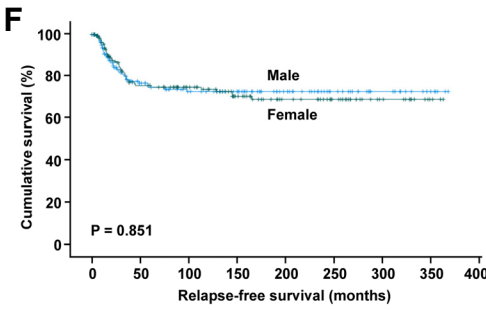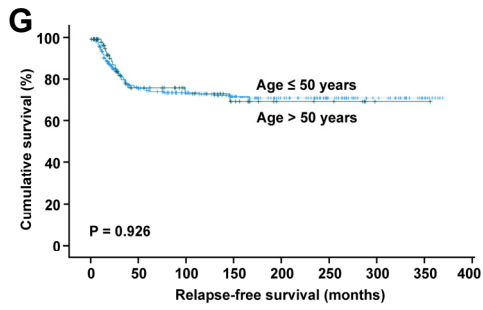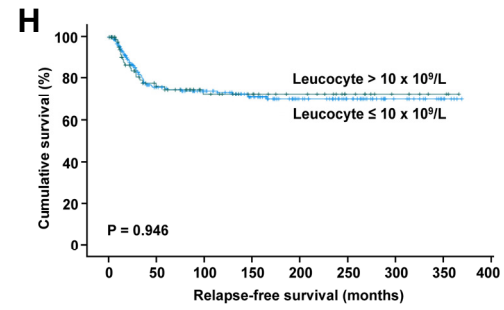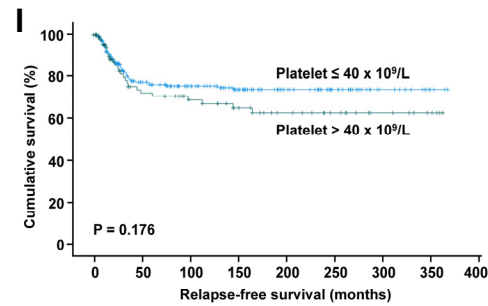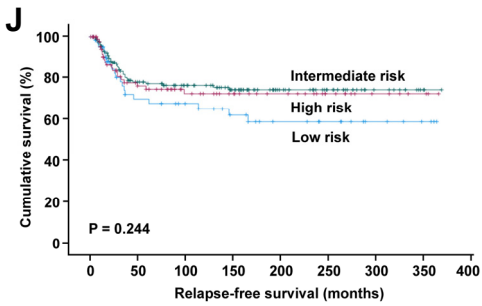

Supplement: Supplementary file 6 — Supplementary Material 6 [file 12885_2023_10612_MOESM6_ESM.pdf]

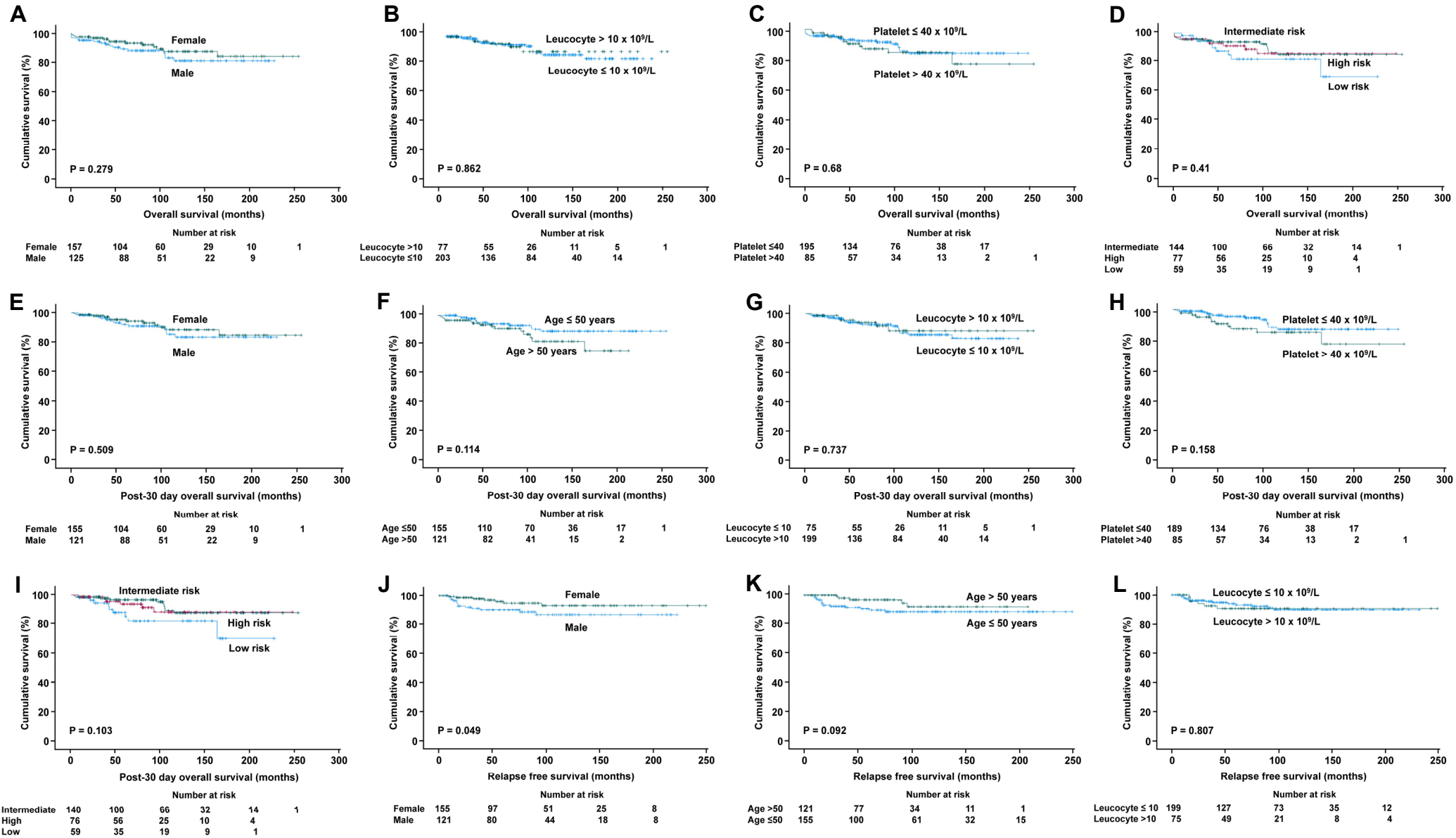

M

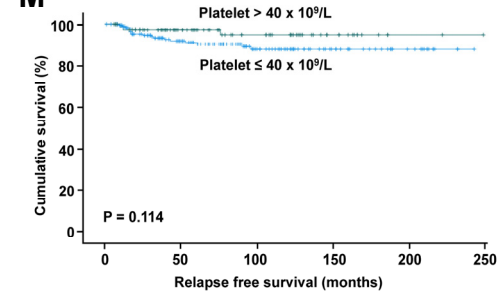

|                |     |     |    |    |    |
|----------------|-----|-----|----|----|----|
| Number at risk |     |     |    |    |    |
| Platelet >40   | 85  | 55  | 33 | 13 | 2  |
| Platelet ≤40   | 189 | 121 | 61 | 30 | 14 |

N

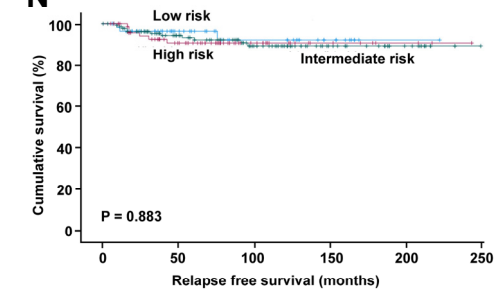

|                |     |    |    |    |    |
|----------------|-----|----|----|----|----|
| Number at risk |     |    |    |    |    |
| Low            | 59  | 33 | 18 | 9  | 1  |
| Intermediate   | 140 | 93 | 56 | 27 | 12 |
| High           | 75  | 50 | 20 | 7  | 3  |

Supplement: Supplementary file 7 — Supplementary Material 7 [file 12885_2023_10612_MOESM7_ESM.pdf]
