# Supplementary material for: Acute promyelocytic leukaemia: population-based study of epidemiology and outcome with ATRA and oral-ATO from 1991 to 2021
Source: BMC Cancer. 2023 Feb 10;23:141. doi: 10.1186/s12885-023-10612-z (PMC9921648; doi:10.1186/s12885-023-10612-z)
Supplement: Supplementary file 4 — Supplementary Material 4 [file 12885_2023_10612_MOESM4_ESM.pdf]

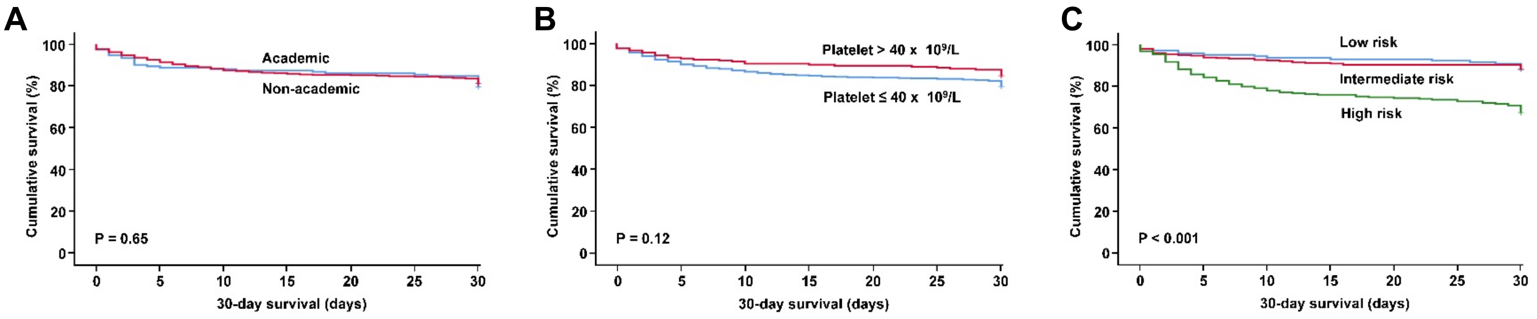

Number at risk

Academic	151	135	133	132	130	130	128
Non-academic	600	555	530	517	512	508	501

Number at risk

Platelet > 40	216	193	189	187	185	184	181
Platelet ≤ 40	519	482	460	448	443	440	434

Number at risk

Low	142	136	134	133	132	131	129
Intermediate	340	322	316	310	307	307	307
High	253	217	200	192	189	186	179
