# Supplementary material for: Acute promyelocytic leukaemia: population-based study of epidemiology and outcome with ATRA and oral-ATO from 1991 to 2021
Source: BMC Cancer. 2023 Feb 10;23:141. doi: 10.1186/s12885-023-10612-z (PMC9921648; doi:10.1186/s12885-023-10612-z)
Supplement: Supplementary file 5 — Supplementary Material 5 [file 12885_2023_10612_MOESM5_ESM.pdf]

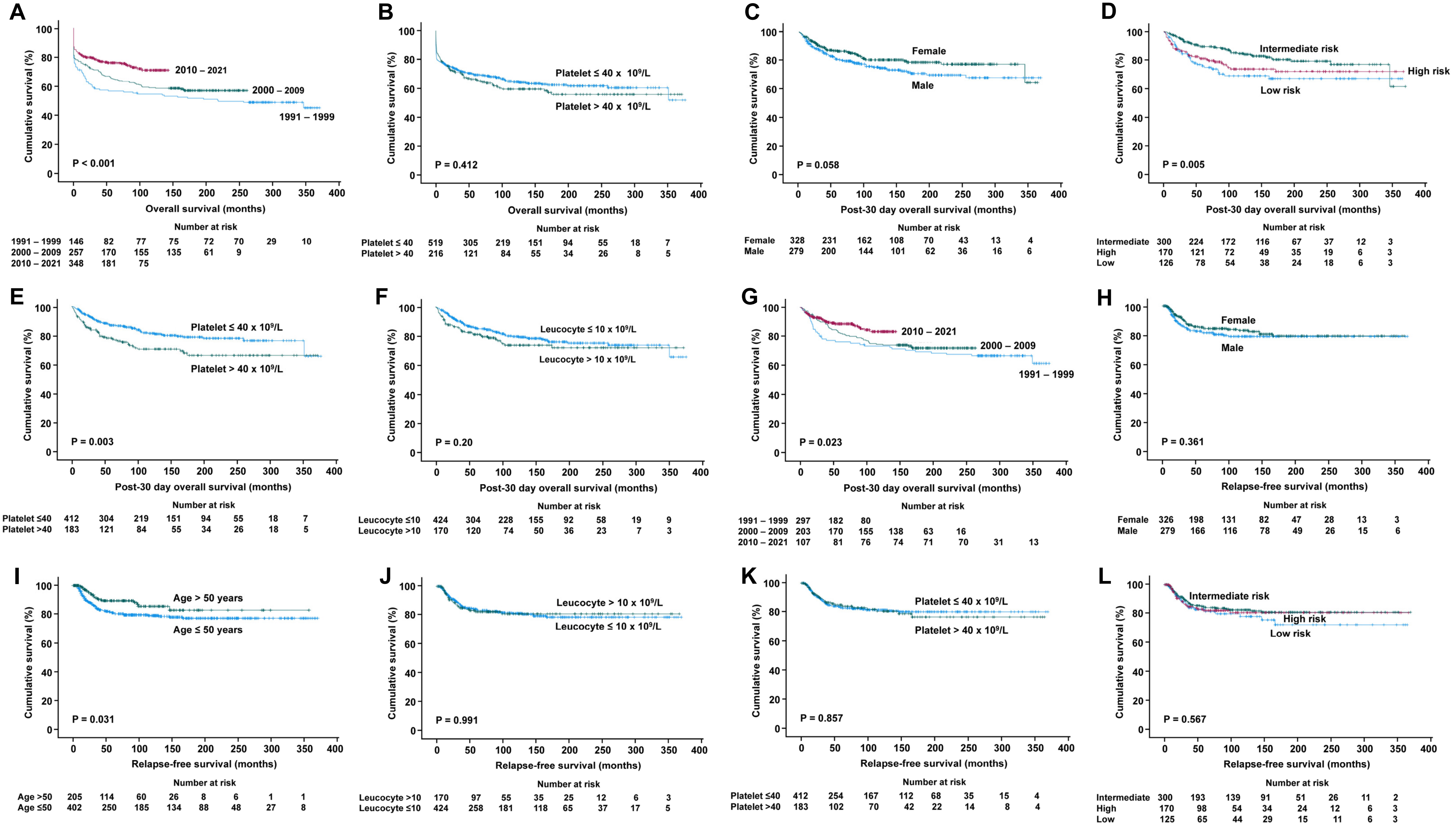
A
Cumulative survival (%)
2010 – 2021
2000 – 2009
1991 – 1999
P < 0.001
Overall survival (months)
Number at risk
1991 – 1999 146 82 77 75 72 70 29 10
2000 – 2009 257 170 155 135 61 9
2010 – 2021 348 181 75
B
Cumulative survival (%)
Platelet ≤ 40 x 10⁹/L
Platelet > 40 x 10⁹/L
P = 0.412
Overall survival (months)
Number at risk
Platelet ≤ 40 519 305 219 151 94 55 18 7
Platelet > 40 216 121 84 55 34 26 8 5
C
Cumulative survival (%)
Female
Male
P = 0.058
Post-30 day overall survival (months)
Number at risk
Female 328 231 162 108 70 43 13 4
Male 279 200 144 101 62 36 16 6
D
Cumulative survival (%)
Intermediate risk
High risk
Low risk
P = 0.005
Post-30 day overall survival (months)
Number at risk
Intermediate 300 224 172 116 67 37 12 3
High 170 121 72 49 35 19 6 3
Low 126 78 54 38 24 18 6 3
E
Cumulative survival (%)
Platelet ≤ 40 x 10⁹/L
Platelet > 40 x 10⁹/L
P = 0.003
Post-30 day overall survival (months)
Number at risk
Platelet ≤40 412 304 219 151 94 55 18 7
Platelet >40 183 121 84 55 34 26 18 5
F
Cumulative survival (%)
Leucocyte ≤ 10 x 10⁹/L
Leucocyte > 10 x 10⁹/L
P = 0.20
Post-30 day overall survival (months)
Number at risk
Leucocyte ≤10 424 304 228 155 92 58 19 9
Leucocyte >10 170 120 74 50 36 23 7 3
G
Cumulative survival (%)
2010 – 2021
2000 – 2009
1991 – 1999
P = 0.023
Post-30 day overall survival (months)
Number at risk
1991 – 1999 297 182 80
2000 – 2009 203 170 155 138 63 16
2010 – 2021 107 81 76 74 71 70 31 13
H
Cumulative survival (%)
Female
Male
P = 0.361
Relapse-free survival (months)
Number at risk
Female 326 198 131 82 47 28 13 3
Male 279 166 116 78 49 26 15 6
I
Cumulative survival (%)
Age > 50 years
Age ≤ 50 years
P = 0.031
Relapse-free survival (months)
Number at risk
Age >50 205 114 60 26 8 6 1 1
Age ≤50 402 250 185 134 88 48 27 8
J
Cumulative survival (%)
Leucocyte > 10 x 10⁹/L
Leucocyte ≤ 10 x 10⁹/L
P = 0.991
Relapse-free survival (months)
Number at risk
Leucocyte >10 170 97 55 35 25 12 6 3
Leucocyte ≤10 424 258 181 118 65 37 17 5
K
Cumulative survival (%)
Platelet ≤ 40 x 10⁹/L
Platelet > 40 x 10⁹/L
P = 0.857
Relapse-free survival (months)
Number at risk
Platelet ≤40 412 254 167 112 68 35 15 4
Platelet >40 183 102 70 42 22 14 8 4
L
Cumulative survival (%)
Intermediate risk
High risk
Low risk
P = 0.567
Relapse-free survival (months)
Number at risk
Intermediate 300 193 139 91 51 26 11 2
High 170 98 54 34 24 12 6 3
Low 125 65 44 29 15 11 6 3
