# Supplementary material for: Acute promyelocytic leukaemia: population-based study of epidemiology and outcome with ATRA and oral-ATO from 1991 to 2021
Source: BMC Cancer. 2023 Feb 10;23:141. doi: 10.1186/s12885-023-10612-z (PMC9921648; doi:10.1186/s12885-023-10612-z)
Supplement: Supplementary file 8 — Supplementary Material 8 [file 12885_2023_10612_MOESM8_ESM.pdf]

Supplemental file 8. Second malignancies in 21 patients with acute promyelocytic leukaemia (APL)

	Acute promyelocytic leukaemia treatment		Second cancer		
Sex	First line	Second line and beyond	Age at onset (years)	Histology	Years after APL
F	Induction: ATRA-daunorubicin<br>Consolidation: daunorubicin+AraC<br>Maintenance: ATRA-6MP-oral MTX	No	52	Endometrial cancer	24
M	Induction: ATRA-daunorubicin<br>Consolidation: daunorubicin+AraC<br>Maintenance: ATRA-6MP-oral MTX	Re-induction: ATRA-idarubicin<br>Autologous HSCT	39	Oesophageal carcinoma	16
F	Induction: ATRA-daunorubicin<br>Consolidation: daunorubicin+AraC<br>Maintenance: ATRA-6MP-oral MTX	No	44	Breast cancer	20
F	Induction: ATRA-daunorubicin<br>Consolidation: daunorubicin+AraC<br>Maintenance: ATRA-6MP-oral MTX	Re-induction: AAA + idarubicin<br>Consolidation: AAA + idarubicin<br>Maintenance: AAA	37	Thyroid papillary carcinoma	15
F	Induction: ATRA-daunorubicin<br>Consolidation: daunorubicin+AraC<br>Maintenance: ATRA-6MP-oral MTX	Re-induction: AAA + idarubicin<br>Consolidation: AAA + idarubicin<br>Maintenance: AAA	45	Endometrial carcinoma	19
F	Induction: ATRA-daunorubicin<br>Consolidation: daunorubicin+AraC<br>Maintenance: ATRA-6MP-oral MTX	No	50	Myelodysplastic syndrome	6
F	Induction: ATRA-daunorubicin<br>Consolidation: daunorubicin+AraC<br>Maintenance: ATRA-6MP-oral MTX	Re-induction: AAA + idarubcini<br>Consolidation: AAA + idarubicin<br>Maintenance: AAA	68	Breast cancer	17
F	Induction: ATRA-daunorubicin<br>Consolidation: daunorubicin+AraC<br>Maintenance: AAA	No	44	Nasopharyngeal cancer	1
M	Induction: ATRA-daunorubicin<br>Consolidation: daunorubicin+AraC<br>Maintenance: AAA	Re-induction: AAA + idarubicin<br>Consolidation: AAA + idarubicin<br>Maintenance: AAA	45	Parotid cancer	1
F	Induction: ATRA-daunorubicin<br>Consolidation: daunorubicin+AraC<br>Maintenance: AAA	No	67	Transitional cell carcinoma of ureters	12

F	Induction: ATRA-daunorubicin<br>Consolidation: daunorubicin+AraC<br>Maintenance: ATRA-6MP-oral MTX	No	58	Lung cancer	3
F	Induction: ATRA-daunorubicin<br>Consolidation: daunorubicin+AraC<br>Maintenance: ATRA-6MP-oral MTX	No	54	Breast cancer	13
F	Induction: ATRA-daunorubicin<br>Consolidation: daunorubicin+AraC<br>Maintenance: AAA	No	47	Colorectal cancer	1
M	Induction: ATRA-daunorubicin<br>Consolidation: daunorubicin+AraC<br>Maintenance: ATRA-6MP-oral MTX	First re-inductio: AAA + idarubicin<br>Second re-induction: AAA + mitoxantrone<br>Autologous HSCT	59	Myelodysplastic syndrome	8
F	Induction: ATRA-daunorubicin<br>Consolidation: daunorubicin+AraC<br>Maintenance: AAA	No	57	Breast cancer	9
F	Induction: ATRA-daunorubicin<br>Consolidation: daunorubicin+AraC<br>Maintenance: ATRA-6MP-oral MTX	Re-induction: AAA + idarubicin<br>Consolidation: AAA + idarubicin<br>Maintenance: AAA	44	Colorectal cancer	2
M	Induction: ATRA-daunorubicin<br>Consolidation: daunorubicin+AraC<br>Maintenance: AAA	No	57	Renal cell carcinoma	5
M	Induction: ATRA-daunorubicin<br>Consolidation: daunorubicin+AraC<br>Maintenance: ATRA-6MP-oral MTX	No	88	Cholangiocarcinoma	20
M	Induction: ATRA-daunorubicin<br>Consolidation: daunorubicin+AraC<br>Maintenance: ATRA-6MP-oral MTX	No	70	Carcinoma of tongue	3
M	Induction: ATRA-daunorubicin<br>Consolidation: daunorubicin+AraC<br>Maintenance: AAA	No	77	Colorectal carcinoma	4
F	Induction: ATRA-daunorubicin<br>Consolidation: daunorubicin+AraC<br>Maintenance: AAA	No	62	Breast cancer	2

M: male; F: female; ATRA: all-trans retinoic acid; Ara-C: cytarabine; 6MP: 6-mercaptopurine; MTX: methotrexate; AAA: oral arsenic trioxide, ATRA, ascorbic acid; HSCT: haematopoietic stem cell transplantation
