# Supplementary material for: Acute promyelocytic leukaemia: population-based study of epidemiology and outcome with ATRA and oral-ATO from 1991 to 2021
Source: BMC Cancer. 2023 Feb 10;23:141. doi: 10.1186/s12885-023-10612-z (PMC9921648; doi:10.1186/s12885-023-10612-z)
Supplement: Supplementary file 9 — Supplementary Material 9 [file 12885_2023_10612_MOESM9_ESM.pdf]

Supplemental file 9. Standardized incidence ratio of second cancers and incidence rate ratio of second cancers in patients with acute promyelocytic leukaemia.

	No. of APL	No. with 2º cancers	Expected No. of APL	SIR of 2º cancers (95% C.I.)	IRR (95% C.I.)[A]
**Total**	678	21	578.08	3.63 (2.32, 5.59)	
**Age (years)***					
<18	60	0	97.94	0.00 (0.00, 4.70)	Reference
18–39	217	5	222.15	2.25 (0.83, 5.46)	undefined
40–59	262	12	217.07	5.53 (3.02, 9.69)	undefined
60–79	122	4	101.83	3.93 (1.27, 10.33)	undefined
≥80	17	0	10.62	0.00 (0.00, 32.99)	undefined
**Sex**					
Male	330	6	319.54	1.88 (0.77, 4.24)	Reference
Female	348	15	330.07	4.54 (2.66, 7.55)	2.42 (0.94, 6.24)
**Period**					
1991– 1999	146	8	134.64	5.94 (2.79, 11.75)	Reference
2000–2009	257	12	230.17	5.21 (2.85, 9.16)	0.88 (0.36, 2.15)
2010–2018[B]	275	1	206.26	0.48 (0.03, 3.09)	0.08 (0.01, 0.65)
**Exposure to oral-ATO at any time**					
No	369	8	369.39	2.17 (1.01, 4.39)	Reference
Yes	309	13	280.22	4.64 (2.60, 7.99)	2.14 (0.89, 5.17)
**Time of exposure to oral-ATO**					
Never	369	8	369.39	2.17 (1.01, 4.39)	Reference
First-line	212	7	188.29	3.72 (1.64, 7.82)	1.72 (0.62, 4.73)
Second-line only	78	5	75.19	6.65 (2.47, 15.49)	3.07 (1.00, 9.39)

APL: acute promyelocytic leukaemia; ATO: arsenic trioxide; No.: number; 2^0: second; C.I.: confidence intervals; SIR: standardized incidence ratio; IRR: incidence rate ratio; A: values labelled as undefined, owing to the fact that IRR cannot be defined in different subgroups of age due to the small sample size; B: The last year where a second primary cancer occurred was in 2018. Comparison against the standard population was therefore until 2018. 2019-2021 was not included for second cancer comparison as the follow-up time for patients was too short to conclude if the incidence of second cancers was increased or decreased
