# Supplementary material for: Acute promyelocytic leukaemia: population-based study of epidemiology and outcome with ATRA and oral-ATO from 1991 to 2021
Source: BMC Cancer. 2023 Feb 10;23:141. doi: 10.1186/s12885-023-10612-z (PMC9921648; doi:10.1186/s12885-023-10612-z)
Supplement: Supplementary file 10 — Supplementary Material 10 [file 12885_2023_10612_MOESM10_ESM.pdf]

Supplemental file 10. Incidence rate ratio of second cancers in acute promyelocytic leukaemia compared to cancer incidence in the general population.

	No.	No. with 2º cancer	No. of cancers in population[A]	Population[A]	IR of cancer (95% C.I)[B]	P-value[C]	Cancer, APL > population	IRR (APL vs population) (95% C.I.)
**Total**	678	21	665,894	181,374,978	254 (253, 255)	< 0.001	Yes	14.3 (9.3, 22.0)
**Age (years)**								
<18	60	0	4,990	58,419,166	14.6 (14.3, 14.9)	0.50	No	undefined[D]
18–39	217	5	41,083	64,307,835	56.6 (56.1, 57.2)	< 0.001	Yes	39.8 (16.5, 95.5)
40–59	262	12	198,136	42,708,670	357 (355, 358)	< 0.001	Yes	15.5 (8.8, 27.3)
60–79	122	4	314,000	19,962,291	1,146 (1142, 1,151)	0.02	Yes	3.43 (1.29, 9.13)
≥80	17	0	107,685	2,999,997	1,961 (1,946, 1,977)	0.50	No	undefined[D]
**Gender**								
Male	330	6	355,849	91,849,843	282 (281, 283)	< 0.001	Yes	6.66 (2.99, 14.82)
Female	348	15	310,045	96,548,117	234 (233, 235)	< 0.001	Yes	19.4 (11.7, 32.2)
**Period**								
1991–1999	146	8	167,371	55,627,198	274 (273, 275)	< 0.001	Yes	21.69 (10.84, 43.36)
2000–2009	257	12	229,847	68,095,442	248 (247, 249)	< 0.001	Yes	21.04 (11.95, 37.04)
2010–2018[E]	275	1	268,676	57,652,338	250 (249, 252)	0.50	No	1.94 (0.27, 13.76)
**Exposure to oral-ATO**								
No	369	8	665,894	181,374,978	254 (253, 255)	< 0.001	Yes	8.5 (4.3, 17.1)
Yes	309	13	665894	181,374,978	254 (253, 257)	< 0.001	Yes	18.3 (10.6, 31.5)
**Time of exposure to oral-ATO**								
Never	369	8	665894	181,374,978	253.9 (253.1, 254.6)	< 0.001	Yes	8.5 (4.3, 17.1)
First-line	212	7	665894	181,374,978	253.9 (253.1, 254.6)	< 0.001	Yes	14.6 (7.00, 30.7)
Second-line only	78	5	665894	181,374,978	253.9 (253.1, 254.6)	< 0.001	Yes	26.2 (10.9, 62.9)

APL: acute promyelocytic leukaemia; ATO: arsenic trioxide; No.: number; 2^0: second; C.I.: confidence intervals; IR: incidence ratio; IRR: incidence rate ratio; A: The World WHO (2000-2025) standard population as the reference standard; B: IR was defined as number of events divided by the person-time at risk; C: Two sample Z test of proportions; D: values labelled as undefined, owing to the fact that IRR cannot be defined in different subgroups of age due to the small sample size; E: The last year where a second primary cancer occurred was in 2018. Comparison against the standard population was therefore until 2018. 2019-2021 was not included for second cancer comparison as the follow-up time for patients was too short to conclude if the incidence of second cancers was increased or decreased
