# Supplementary material for: Acute promyelocytic leukaemia: population-based study of epidemiology and outcome with ATRA and oral-ATO from 1991 to 2021
Source: BMC Cancer. 2023 Feb 10;23:141. doi: 10.1186/s12885-023-10612-z (PMC9921648; doi:10.1186/s12885-023-10612-z)
Supplement: Supplementary file 11 — Supplementary Material 11 [file 12885_2023_10612_MOESM11_ESM.docx]

**Supplemental files**

Supplemental file 1. Treatment protocols for newly-diagnosed APL

Supplemental file 2. Calculation of standardized incidence ratio (SIR) of second primary cancers in APL patients

Supplemental file 3. Epidemiology of APL. A: number of patients per calendar year; B: breakdown of early deaths

Supplemental file 4. Impact of various clinicopathologic parameters on 30-day survivals. These parameters were not significant on multivariate analysis

Supplemental file 5. Impact of various clinicopathologic parameters on survivals. These parameters were not significant on multivariate analysis

Supplemental file 6. Impact of various clinicopathologic parameters on survivals of patients treated with ATRA-based regimens. These parameters were not significant on multivariate analysis

Supplemental file 7. Impact of various clinicopathologic parameters on survivals of patients treated with oral-ATO-based regimens. These parameters were not significant on multivariate analysis

Supplemental file 8. Second malignancies in 21 patients with APL

Supplemental file 9. Standardized incidence ratio of second cancers and incidence rate ratio of second cancers in patients with APL.

Supplemental file 10. Incidence rate ratio of second cancers in acute promyelocytic leukaemia compared to cancer incidence in the general population.
